# Discharge Perfection: Assessing Documentation Quality at Mardan Medical Complex, Pakistan (2024 Audit Debut)

**DOI:** 10.7759/cureus.65625

**Published:** 2024-07-29

**Authors:** Murad Ali, Manzoor Hussain, Muhammad Mushtaq, Amjad Ali, Hameed Zaib

**Affiliations:** 1 Medicine, Bacha Khan Medical College, Mardan, PAK; 2 General Medicine, Mardan Medical Complex, Mardan, PAK; 3 Internal Medicine, Mardan Medical Complex, Mardan, PAK; 4 Monitoring and Quality Assurance, Mardan Medical Complex, Mardan, PAK

**Keywords:** quality indicator, medical ward, clinical audit, discharge documentation, mardan medical complex, communication tools, quality improvement, electronic medical records (emr), discharge summary, clinical documentation audit

## Abstract

Background

Patient discharge summaries not only play a vital role in ensuring continuity of care and patient safety but also serve as a communication tool between the primary and tertiary care settings. However, despite their paramount importance, most discharge summaries are either inaccurate or miss essential clinical information, posing considerable danger to patients. This clinical audit assesses the quality of discharge summaries at Mardan Medical Complex, Mardan, Pakistan, to identify areas for improvement.

Aim

The aim of this study is to assess the discharge summaries of patients at Mardan Medical Complex in Mardan, Pakistan, with a focus on their completeness, accuracy, and timeliness.

Methods

A cross-sectional, observational, and retrospective study was carried out in the Medical A ward of Mardan Medical Complex, Mardan, Pakistan, from September 2023 to October 2023. Out of the 897 discharge slips, a sample size of 105 participants was determined using Epi Info software. A systematic random sampling technique was used. Data was extracted from the hospital management information system and evaluated using a clinical audit tool based on standard guidelines from the Royal College of Physicians, Islamabad Healthcare Regulatory Authority, and Khyber Pakhtunkhwa Health Care Commission. To analyze the data, descriptive statistics were applied.

Results

The clinical audit revealed significant deficiencies in discharge summaries. Important patient demographics, such as contact details and safety alerts, were completely absent in 100% of the cases, and 48% of the summaries lacked the father’s name. Admission details were similarly inadequate, with nearly all summaries missing critical information like admission time and reasons for admission. Clinical summaries and procedural details were absent in 73% and 87% of the cases, respectively. Discharge planning also showed major gaps, as special instructions according to the primary diagnosis and discharge destination were frequently neglected. Follow-up visits were recommended in only 71% of cases. Additionally, there were significant errors in in-home medication prescriptions, with 61% missing medication doses, 28% missing the route of administration, and 20% lacking the duration of treatment.

Conclusions

This clinical audit identified critical areas for improvement by revealing significant errors in the quality of discharge summaries at Mardan Medical Complex. It is recommended that standardized discharge slip templates be implemented, healthcare workers receive proper training, and thorough monitoring be conducted before patients are discharged. These measures aim to enhance the standard of documentation. Additionally, regular future clinical audits are essential for tracking the impact of these interventions and ensuring patient safety and continuity of care.

## Introduction

A hospital discharge summary provides comprehensive details of a patient’s hospitalization journey, including demographics, diagnosis, treatments, home medications, and follow-up instructions necessary for ensuring continuity of care after leaving the hospital [1]. This important document is usually issued in the form of an electronic discharge summary at the time of the patient’s departure from the hospital. It serves as the primary source of communication between tertiary and primary medical care [2]. Therefore, it is necessary that this document be precise and complete in all aspects to transfer all the essential information regarding patient care to ensure patient safety and uniformity of care between the hospital and community [3]. It has been demonstrated that patients may suffer negative consequences if the discharge summaries carry inaccurate and inadequate information. These consequences may include a higher risk for rehospitalization, problems from medication errors, and increased morbidity and mortality [4].

Despite their crucial role, most discharge summaries frequently remain incorrect and incomplete in most parts of the world [5]. In the United States, up to 33% of discharge summaries remain deficient in vital patient information [6], while in Europe, these figures soar to a shocking 40% [7]. In Pakistan, the quality of discharge summaries varies greatly, from being accurate to awfully incomplete. A recent study carried out at a tertiary care hospital in Pakistan highlighted drastic errors needing urgent improvement. Having the same format, 16% of the discharge summaries were difficult to read, and crucial patient information like comorbidities and procedural details were missing, putting patient safety and care continuity at risk [8].

In response to these obvious shortcomings, our institution embarks on a clinical audit to assess the overall quality of our patients’ discharge summaries. By carefully examining the completeness, correctness, and timeliness, our main aim is to identify areas for improvement and implement focused interventions. Our goal is crystal clear: to elevate the quality of our hospital discharge documentation from a potential risk to a cornerstone of clinical excellence and patient-centered care.

## Materials and methods

We conducted a cross-sectional, observational, and retrospective study from September 2023 to October 2023 in the Medical A ward of Mardan Medical Complex, Mardan, Pakistan, to assess the quality of discharge slips. The sample size calculated was 105 participants using the Epi Info software to ensure the statistical reliability of our study findings. The total discharge summaries in the two months from September to October 2023 were 897, and taking a 95% confidence interval and a margin of error set at 9.5%, this sample size was quite sufficient to provide meaningful insights into the audit objectives while maintaining statistical accuracy.

The discharge summaries were chosen from a sampling frame using systematic random sampling techniques at a sampling interval of 8. To determine this sampling interval, the total population of discharge summaries (n = 897) was divided by the sample size (n = 105).

**Figure 1:**
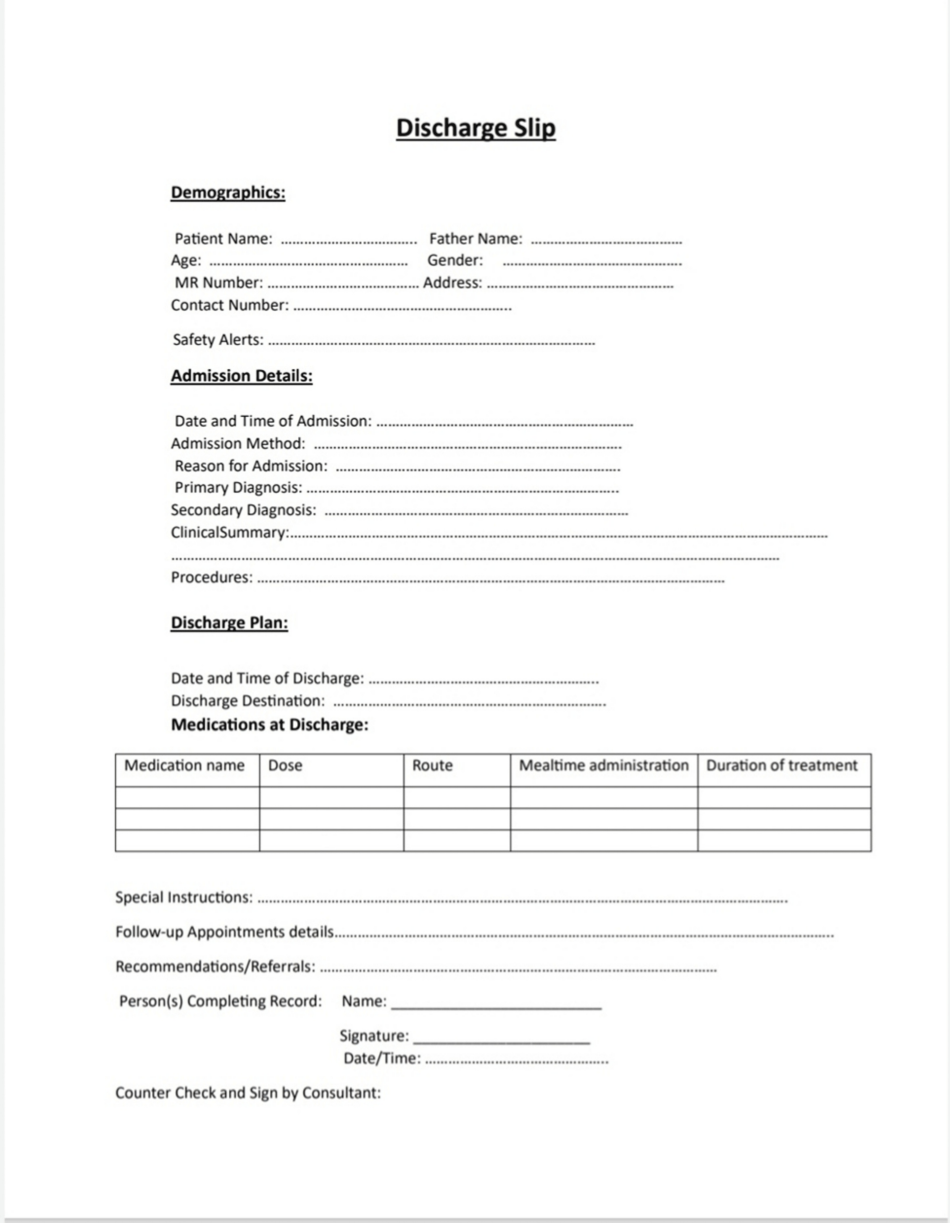
Audit tool for the assessment of discharge slips at Mardan Medical Complex

The hospital management information system of Mardan Medical Complex was used for meticulous data retrieval for this study. We carefully designed an audit tool (Figure 1) for our evaluation of selected discharge summaries, taking insights from well-reputed international bodies such as the Royal College of Physicians [9], as well as national entities like the Islamabad Healthcare Regulatory Authority [10] and the local Khyber Pakhtunkhwa Health Care Commission’s minimum healthcare delivery standards [11]. This was a comprehensive tool containing a range of criteria tailored to assess critical aspects of a discharge summary, with a particular emphasis on patient demographics, admission details, and discharge planning components.

Each selected discharge slip was evaluated against the criteria outlined in the audit tool, ensuring meticulous scrutiny of each component. Subsequently, comprehensive data was collected to assess the quality of selected discharge summaries.

The collected data was analyzed using simple statistical methods. For assessing the filling and completeness of discharge summary information, descriptive statistics such as percentages and frequencies were calculated through Microsoft Excel 2013 software (Microsoft Corporation, Redmond, United States). To provide a clear overview of the evaluation outcomes, the results were visually illustrated in the form of tables and figures.

This simple statistical approach was sufficient to provide a clear assessment of the accuracy, completeness, and adherence to standard guidelines in discharge documentation.

## Results

The evaluation of discharge slip documentation quality at Mardan Medical Complex unveiled a landscape of moderate quality, revealing significant gaps in crucial information. Initial observations highlighted a lack of organization in presenting various parameters, leading to patient confusion.

**Figure 2:**
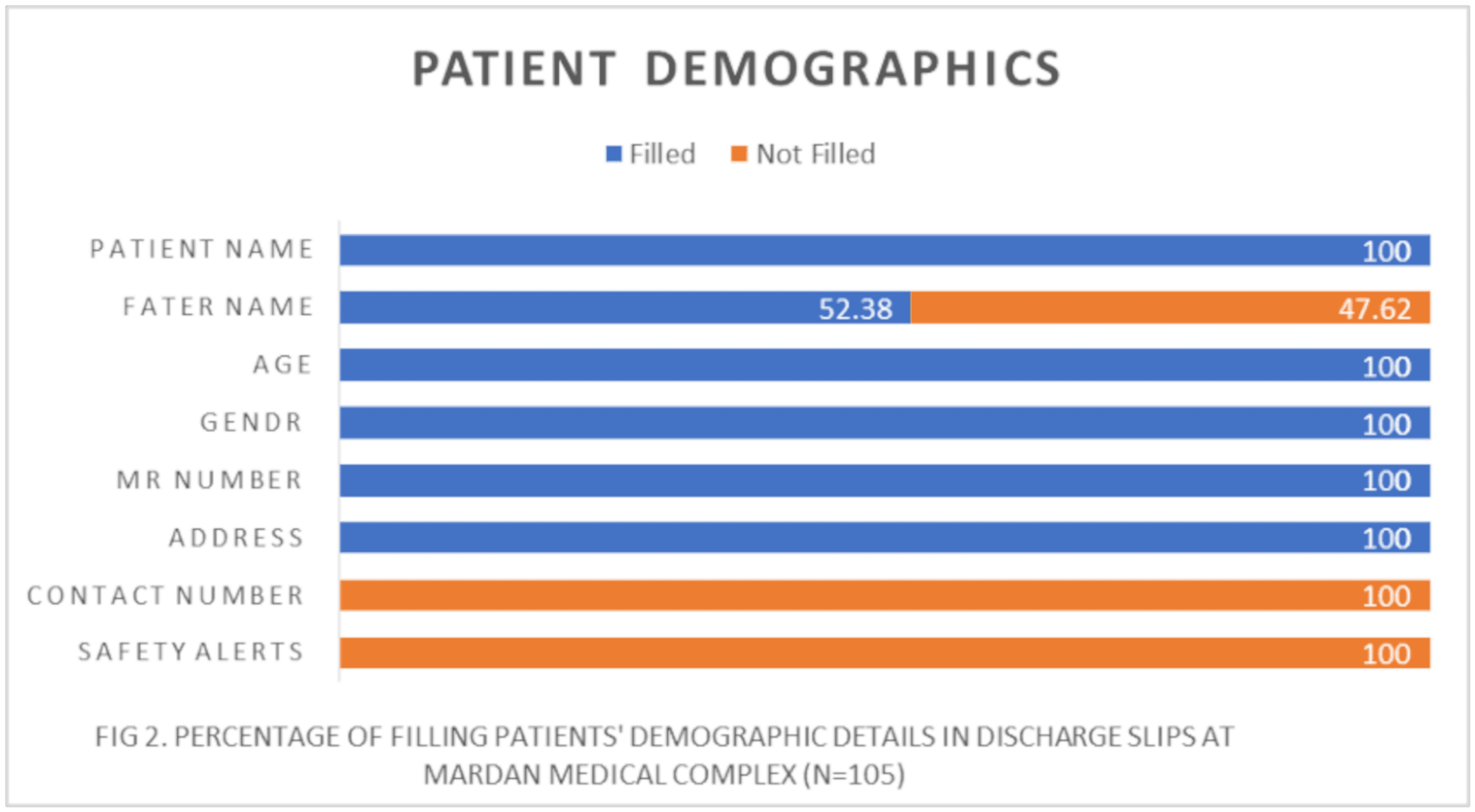
Percentage of filling patients’ demographic details in discharge slips at Mardan Medical Complex (n = 105)

Concerning patient demographics, glaring omissions were evident, with vital contact details and safety alerts conspicuously absent, registering at a disconcerting “0%” on the assessment graph. Additionally, nearly half of the discharge slips (48%) failed to include the patient’s father’s name, masking their identity, which raises serious privacy concerns. (Table 1, Figure 2).

**Table 1:** Demographic details of patients in discharge slips at Mardan Medical Complex (n = 105)

Parameters	Standard	Observed
Patient’s name	100%	100
Father’s name	100%	52.38%
Age	100%	100%
Gender	100%	100%
MR number	100%	100%
Address	100%	100%
Contact number	100%	0%
Safety alerts	80%	0%

**Figure 3:**
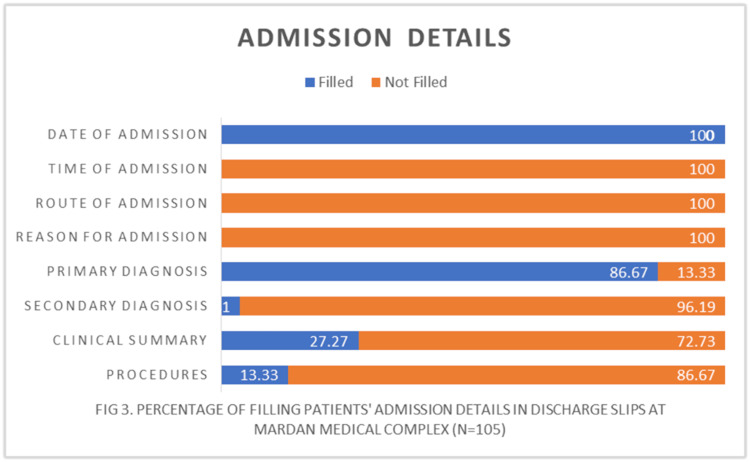
Percentage of filling patients’ admission details in discharge slips at Mardan Medical Complex (n = 105)

Delving into admission details revealed an even more alarming scenario, with critical information notably absent, casting doubt on the accuracy of the records. Essential data such as admission time, reasons for admission, and secondary diagnoses were glaringly missing from nearly all evaluated discharge slips. Furthermore, clinical summaries and procedures were noticeably absent in 73% and 87% of cases, respectively, further exacerbating the documentation deficit (Table 2, Figure 3).

**Table 2:** Admission details of patients’ discharge summaries at Mardan Medical Complex (n = 105)

Parameters	Standard	Observed
Date of admission	100%	100%
Time of admission	100%	0%
Route of admission	100%	0%
Reason for admission	100%	0%
Primary diagnosis	100%	86.67%
Secondary diagnosis	90%	3.81%
Clinical summary	100%	27.27%
Procedures	95%	13.33%

In the discharge plan section, the presence of significant errors underscored the urgent need for improvement. Discharge destination and special instructions were notably omitted, while the absence of counterchecking and signatures by consultants raised doubts about the validity of the information provided. Although the completion of records by designated personnel was documented in 41% of cases, follow-up appointments were only advised in 71% of instances, indicating inconsistent practices.

**Figure 4:**
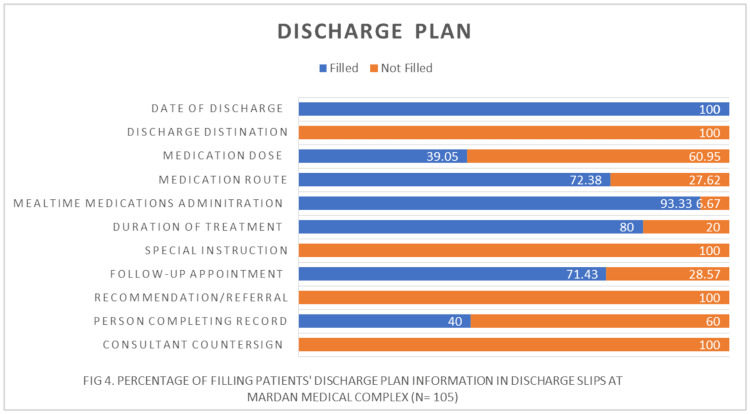
Percentage of filling patients' discharge plan information in discharge slips at Mardan Medical Complex (n = 105)

Of particular concern were errors in in-home medication prescriptions, with substantial gaps in medication dose (61% missing), medication route (28% missing), and treatment duration (20% missing). These discrepancies highlight potential risks to patient safety and the need for comprehensive improvement measures in discharge documentation protocols (Table 3, Figure 4).

**Table 3:** Discharge plan information in discharge summaries of patients at Mardan Medical Complex (n = 105)

Parameters	Standard	Observed
Date of discharge	100%	100%
Discharge destination	90%	0%
Medication dose	100%	39.50%
Medication route	100%	72.38%
Duration of treatment	95%	80%
Mealtime medication administration	95%	93.33%
Follow-up appointment	95%	71.42%
Special instructions	95%	0%
Recommendations/referrals	90%	0%
Person completing record	100%	60%
Consultant countersign	95%	0%

## Discussion

The findings of this clinical audit identified significant deficiencies in the quality of discharge documentation at Mardan Medical Complex, Mardan, Pakistan, which highlights the urgent need for systemic improvements to ensure patient safety and continuity of care.

The absence of essential contact details and safety alerts in the demographic section of discharge summaries is of particular concern, as it poses a significant risk to patient safety after discharge. Similar concerns were noted in an international study by Moore et al., where an increased risk of rehospitalization, complications, and even mortality was noted due to incomplete discharge documentation [4]. Our findings also showed missing patients’ fathers’ names and other critical identifiers in 48% of discharge slips, reflecting the issues of legibility and completeness highlighted in a study a few years ago by La Regina et al. [12].

The absence of crucial admission details, such as time of admission, route of admission, reasons for admission, and secondary diagnoses, in nearly all the evaluated discharge slips raises serious doubts about the accuracy and usefulness of the documents for ongoing patient care. As primary care providers rely heavily on this information, missing it can lead to significant discontinuities in care, as revealed by the high number of readmissions within a short period of time. The absence of clinical summaries and procedural details in 73% and 87% of cases, respectively, further exacerbates these concerns, potentially leading to medical errors and adverse patient outcomes [13].

Similarly, crucial information was omitted in the discharge planning section, with notable omissions in discharge destination and special instructions according to primary diagnosis and a lack of counterchecking and consultant signatures. These deficiencies highlight the need for a more structured and reliable discharge planning process. The observed inconsistency in advising follow-up appointments, documented in only 17% of cases, indicates a lack of standardization in discharge protocols, which could jeopardize patient follow-up care and overall health outcomes [14].

The most alarming finding from our audit pertains to the errors in in-home medication prescriptions. With substantial gaps in medication dose (61% missing), medication route (28% missing), and treatment duration (20% missing), patients are at risk of medication errors, which are well-documented causes of adverse health outcomes and hospital readmissions [15,16]. These findings align with international concerns about the quality of discharge documentation and its impact on patient safety [17].

The limitations of our study included a lack of similar studies in our hospital and nearby teaching institutions to aid in designing the study methodology and comparing the findings. Moreover, the study was conducted in just one medical unit of a tertiary care hospital, so it could not be generalizable to other hospitals or medical units. Due to the small sample size in our study, it was difficult to draw definitive conclusions about the relationship between discharge documentation quality and adverse health outcomes. Future research should aim to replicate these findings in larger and more diverse settings to improve generalizability. Additionally, collaboration with other healthcare institutions could help provide a broader perspective on this issue.

## Conclusions

Our audit has identified critical gaps in discharge documentation at Mardan Medical Complex, posing significant risks to patient safety and continuity of care. To address these issues, it is imperative to implement targeted interventions, such as developing a new guidelines-based discharge slip format, training physicians to create standard discharge summaries, and fostering a culture of quality improvement. These measures are essential to enhance patient outcomes and uphold the standards of patient-centered care.

Our institution has implemented the newly designed discharge slip format in the hospital wards, as illustrated in Figure 1, which served as an audit tool. We will conduct a reaudit of the discharge slip quality every six months until we achieve at least 95% compliance with the standards.
